# Chromosome anomalies in bone marrow as primary cause of aplastic or hypoplastic conditions and peripheral cytopenia: disorders due to secondary impairment of *RUNX1* and *MPL* genes

**DOI:** 10.1186/1755-8166-5-39

**Published:** 2012-10-01

**Authors:** Cristina Marletta, Roberto Valli, Barbara Pressato, Lydia Mare, Giuseppe Montalbano, Giuseppe Menna, Giuseppe Loffredo, Maria Ester Bernardo, Luciana Vinti, Simona Ferrari, Alessandra Di Cesare-Merlone, Marco Zecca, Francesco Lo Curto, Franco Locatelli, Francesco Pasquali, Emanuela Maserati

**Affiliations:** 1 Biologia e Genetica, Dipartimento di Medicina Clinica e Sperimentale, Università dell'lnsubria, Varese, Italy; 2 Department of Oncology, Azienda "Santobono-Pausilipon", Pausilipon Hospital, Napoli, Italy; 3 Dipartimento di Onco-Ematologia Pediatrica, Ospedale Bambino Gesù, Roma, Università di Pavia, Pavia, Italy; 4 U.O. Genetica Medica, Policlinico S.Orsola-Malpighi, Bologna, Italy; 5 Oncoematologia Pediatrica, Fondazione IRCCS Policlinico S. Matteo, Pavia, Italy; 6 Dipartimento di Medicina Clinica e Sperimentale, Università dell’lnsubria, Via J. H. Dunant 5, I 21100, Varese, Italy

**Keywords:** SAA, Thrombocytopenia, CAMT, *RUNX1*, *MPL*, Chromosome structural anomalies, Chromosome 1, Chromosome 21

## Abstract

**Background:**

Chromosome changes in the bone marrow (BM) of patients with persistent cytopenia are often considered diagnostic for a myelodysplastic syndrome (MDS). Comprehensive cytogenetic evaluations may give evidence of the real pathogenetic role of these changes in cases with cytopenia without morphological signs of MDS.

**Results:**

Chromosome anomalies were found in the BM of three patients, without any morphological evidence of MDS: 1) an acquired complex rearrangement of chromosome 21 in a boy with severe aplastic anaemia (SAA); the rearrangement caused the loss of exons 2–8 of the *RUNX1* gene with subsequent hypoexpression. 2) a constitutional complex rearrangement of chromosome 21 in a girl with congenital thrombocytopenia; the rearrangement led to *RUNX1* disruption and hypoexpression. 3) an acquired paracentric inversion of chromosome 1, in which two regions at the breakpoints were shown to be lost, in a boy with aplastic anaemia; the *MPL* gene, localized in chromosome 1 short arms was not mutated neither disrupted, but its expression was severely reduced: we postulate that the aplastic anaemia was due to position effects acting both in *cis* and in *trans*, and causing Congenital Amegakaryocytic Thrombocytopenia (CAMT).

**Conclusions:**

A clonal anomaly in BM does not imply *per se* a diagnosis of MDS: a subgroup of BM hypoplastic disorders is directly due to chromosome structural anomalies with effects on specific genes, as was the case of *RUNX1* and *MPL* in the patients here reported with diagnosis of SAA, thrombocytopenia, and CAMT. The anomaly may be either acquired or constitutional, and it may act by deletion/disruption of the gene, or by position effects. Full cytogenetic investigations, including a-CGH, should always be part of the diagnostic evaluation of patients with BM aplasia/hypoplasia and peripheral cytopenias.

## Background

In the clinical practice, the finding of an acquired chromosome change in a patient with persistent peripheral cytopenia and aplastic or hypoplastic bone marrow (BM), in absence of conclusive morphologic features, is usually interpreted as an indicative sign of myelodysplastic syndrome (MDS), although the World Health Organization (WHO) classification of myeloid neoplasms lists only 14 recurrent anomalies as presumptive evidence of MDS [1]. In the years 2000–2011 we performed cytogenetic investigations, as part of routine work, in 87 pediatric patients with persistent cytopenia, either uni-, bi- or trilinear, during their diagnostic evaluation. In this heterogeneous cohort we found monosomy 7 and trisomy 8 in two patients each, all eventually diagnosed as MDS; increased chromosome breakage was observed in three cases then diagnosed as Fanconi anaemia (FA); an isochromosome for the long arms of chromosome 7 was present in one patient with previously undiagnosed Shwachman-Diamond Syndrome; trisomy 8 was found in one patient in whom *MPL* gene mutations demonstrated a congenital amegacaryotic thrombocytopenia (CAMT, OMIM # 604998) [2]; a translocation t(8;17)(p21;q25) was present in a patient with features of Blackfan-Diamond Anaemia. Moreover, in at least three patients out of 87, without any morphological evidence of MDS, a structural chromosome anomaly in the BM was the primary event leading to a specific disease. We report here these three patients, and suggest that this pathogenetic pathway may be rather frequent. Complex structural anomalies of chromosome 21 were present in two of these patients, leading to the disruption or to the loss of the *RUNX1* gene, with decreased expression and different haematological and clinical pictures: severe aplastic anaemia (SAA) and congenital thrombocytopenia. In the third patient, a paracentric inversion of chromosome 1 was present, and we postulate that it led to aplastic anaemia through position effects on the *MPL* gene, with severely reduced expression; this interpretation turned the diagnosis to CAMT.

### Clinical reports

#### Patient 1

Male child, born in 1997 from non-consanguineous parents, who was diagnosed in 2004 with SAA with the following blood counts: RBC 1.66x10^12^/L, Hb 58 g/L, reticulocytes 0.035, platelet 11x10^9^/L, WBC 1.8x10^9^/L with 75.4% neutrophils, 11.2% lymphocytes, 12.7% monocytes, 0.5% eosinophils, 0.2% basophils. BM biopsy showed decreased cellularity (10%) with a picture of severe hypoplasia affecting in particular the granulocytic and megakaryocytic lineages. Glycoforin and myeloperoxidase immunostaining showed fair conservation of the erythroid series and severe scarcity of the granulopoietic one. The diepoxybutane (DEB) test excluded FA. Two cycles of immunosuppressive therapy were administered in February and August 2004, according to the protocol “EBMT SAA Working Party” consisting of anti-lymphocyte globulin, cyclosporine A, prednisone, and granulocyte-colony stimulating factor (G-CSF). A slow, progressive improvement was obtained, with no further need for transfusions. In August 2006 the patient did fairly well and his blood counts were as follows: RBC 3.79x10^12^/L, Hb 121 g/L, platelet 88x10^9^/L, WBC 2.9x10^9^/L, with 56.4% neutrophils, 32.2% lymphocytes, 0.3% monocytes, 0.1% eosinophils, 0 basophils. BM cellularity was increased, with particular regard to the granulocytic and megakaryocytic components. The platelet morphology and function were normal, except for the presence of spontaneous platelet aggregation. From 2007 to 2009 he remained in fairly good health notwithstanding a progressive decrease of Hb, RBC, WBC and platelet values. At the beginning of 2009, the patient became gain dependent on platelet and erythrocyte transfusion. In June 2009, an HLA-identical unrelated donor was found and the patient underwent transplantation of haematopoietic stem cells (HSCT) a month later, after receiving a conditioning regimen including fludarabine and low-dose cyclophosphamide. The allograft was rejected and a second transplant was performed, employing the HLA-partially matched mother as donor. The patient was given a T-depleted allograft, consisting of positively selected CD34+ cells after a preparative regimen including treosulfan and fludarabine. This second allograft resulted in an engraftment of donor cells, although the haematopoietic recovery was incomplete as the patient remained dependent of platelet transfusion and the neutrophil count did not exceed 0.20x10^9^/L. For this reason, a second infusion of positively selected CD34+ cells was performed in February 2010 without any preparative regimen. After this third allograft, the patient recovered normal cell blood counts and he is now alive, in complete donor chimerism without any sign of graft-versus-host disease.

#### Patient 2

Female child, born in 2000 from non-consanguineous parents, who was hospitalized due to thrombocytopenia discovered at 11 days of life (platelets 54x10^9^/L), and then again confirmed at 7 months. She had a small defect of the ventricular septum, which subsequently closed spontaneously; cow’s milk intolerance was diagnosed. In the course of the years, her thrombocytopenia remained moderate and asymptomatic (e.g. 79x10^9^/L at 7 years, and 91x10^9^/L at 10 years) and was monitored until 10 years of age, being accompanied by mild normocytic anaemia (Hb 100–112 g/L), with normal reticulocyte count, and normal foetal haemoglobin. The examination of a BM smear at 8 years of age showed an almost normal presence of all cell lines, with some reduction of the erythroid series. A comprehensive clinical evaluation failed to reveal any dysmorphisms or other pathological signs.

The DEB test excluded FA. No mutations of the *MPL* and *RUNX1* genes were present.

#### Patient 3

Male child, born in 2004 from 4^th^ degree consanguineous parents, who was referred for aplastic anaemia. He had an elder sister, born in 2000, in whom a diagnosis of aplastic anaemia was made at 14 months of age in Tunis, Morocco, with blood counts referred as follows: Hb 45 g/L, WBC 3.4x10^9^/L, platelets 7x10^9^/L; no malformative/dysmorphic signs were present; her karyotype has been referred as normal, both on BM and on PB. She died at the age of two years for a severe infection. Another elder sister, born in 1996, is in good health. Then, in 2011, a healthy brother was born.

The child was examined in August 2007, when his blood counts showed: Hb 62 g/L, RBC 2.97x10^12^/L, WBC 2.5x10^9^/L with 13% neutrophils, 83% lymphocytes, 0.4% monocytes, 2.7% eosinophils, 0.4% basophils, platelets 18x10^9^/L. BM trephine biopsy revealed severely reduced cellularity, with a few residual myeloid and erythroid elements and no megakaryocytes (Mks). Eight months later, a further biopsy revealed complete BM aplasia. The DEB test excluded FA. Mutational analysis of the *MPL* gene, extended to the entire gene, was negative. Two polymorphisms in introns of the *MPL* gene (IVS3 130 + 47 C > T, and IVS6 327–41 G > A) were found in homozygosity both in the patient, and in his elder healthy sister. The finding that these two variants were shared in heterozygosity by the consanguineous parents, indicates that both children inherited from the parents the same *MPL* alleles. In the period 2007–2011, the child was treated with haematopoietic growth factors (G-CSF and erythropoietin) and received iron chelation treatment (intra-venous desferoxamine). The trilinear cytopenia remained substantially stable, and the child required regular transfusions of red blood cell and platelet concentrates. His blood tests consistently showed Hb between 70 and 85 g/L, RBC around 2x10^12^/L, WBC 1–2.5x10^9^/L, platelets 15-40x10^9^/L. Serum thrombopoietin (TPO) level, evaluated in February 2010, was greatly increased: 3500 pg/mL (n. v. 6.9-54.4). Platelet surface expression of *MPL*, evaluated as anti-MPL/anti-isotype ratio [3] was decreased: 1.28 (n. v. 3.18 ± 0.39). A combined cord blood and bone marrow HSCT was performed in July 2011, using the HLA-identical brother born in January 2011 as donor. The transplant was followed by complete donor engraftment and the progressive achievement of transfusion-independence and of a completely normal blood count.

## Results

### Patient 1

The chromosome analyses on BM revealed a clonal structural anomaly of chromosome 21, der(21), present since 2004, a few months after the disease onset, up to June 2009, whereas analyses performed at two different dates in 2006 on peripheral blood (PB) phytohaemagglutinin (PHA)-stimulated cultures showed a normal karyotype in 100 mitoses scored. In most analyses on BM the cells with normal karyotype were the majority, and the abnormal cells were few (e.g. 2/20 in February 2006), but this proportion was in fact somehow variable (e.g. 10/17 in October 2005; 8/23 in May 2006). Fluorescent in situ hybridization (FISH) showed the lack of the signal of the probe CTD-2235K24, which recognizes a sequence on chromosome 21 at band q22.12, in the der(21), and this probe was then used on interphase nuclei in cytogenetic monitoring: it revealed the presence of the der(21) also when no mitoses were observed, and showed that the percentage of abnormal cells was 5-50% in the period 2004–2006, and progressively decreased (17-5%) in years 2006–2008. The decreasing of the der(21) clone paralleled the appearance of a second independent clone with an interstitial deletion of the long arms of chromosome 13 as sole chromosome anomaly, int del (13)(q12-13q21). In the years 2007–2009, this clone progressed in size: from 1/12 to 9/15 mitoses, and from 35/604 to 317/979 nuclei, as evaluated by FISH with the probe RP11-1001I7, that recognize sequences at band 13q14.2.

**Figure 1:**
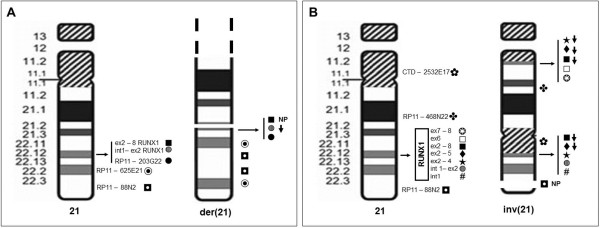
**Ideograms of the normal chromosome 21, and of the rearranged 21s.** The der(21) of patient 1 (**A**), and the inv(21) of patient 2 (**B**), summarizing the most informative FISH results. The following symbols represent the probes related to *RUNX1* sequences used, and the corresponding exons are indicated in the figure, with signals present, not present or weak: # WI2-1915K14, ◍ CTD-2349F18, ★ WI2-942D2, ♦ WI2-605D9, ■ CTD-2235K24, □ WI2-847D7, ❂ WI2-542L2, ⬇ weak signal, NP signal not present. The other BAC probes used and mentioned in the text, but not related to RUNX1 sequences, are shown in the figure with other symbols

The banding pattern of the der(21) was interpreted as a partial duplication of the long arms: the FISH with the library for WCP painted the entire der(21), and the subtelomeric sequences were retained in the expected position as shown by the probe 21qtel07. We started to investigate the *RUNX1* gene by FISH with the probe “LSI AML1-ETO”, designed to detect the translocation t(8;21), recurrent in acute myeloid leukaemia (AML): it revealed a signal of *RUNX1* on the der(21) less intense compared to the normal 21. So we used a panel of BAC probes to investigate the *RUNX1* gene (Table 1). Informative results are shown in Figure 1A: the exons 2–8 of *RUNX1* were absent, the probe recognizing part of the first intron and exon 2 gave a weak signal, while a more distal probe (RP11 – 203 G22) flanking *RUNX1* was normally present, as was the probe RP11-79 G23, mapping at a position more close to the centromere, in 21q21.3 (not shown in the figure). The duplicated region was demonstrated to be also inverted by dual color FISH with two probes mapping in q22.2 and q22.3 (Figure 1A). Thus, the der(21) was in fact the result of a complex rearrangement with a region duplicated and inverted distal to a small interstitial deletion which encompassed most *RUNX1*.

**Table 1:** Probes and libraries used for FISH

**Pt**	**Chromosome**	**Probes/libraries**	**Localization/Sequences recognized**
1	13	RP11-1001I7^a^	13q14.2
	21	WCP 21^b^	whole chromosome paint library
		21qtel07^c^	subtelomeric region
		AML1/ETO^d^	designed for t(8;21)
		RP11-79G23^a^	21q21.3
		RP11-203G22^a^	21q22.12
		CTD-2349F18^a^	*RUNX1* gene, intron1-exon2
		CTD-2235K24^a^	*RUNX1* exons 2–8
		RP11-625E21^a^	21q22.2
		RP11-88N2^a^	21q22.3
2	21	WCP 21^b^	whole chromosome paint library
		21qtel07^c^	subtelomeric region
		AML1/ETO^d^	designed for t(8;21)
		CTD-2532E17^a^	21p11.2-11.1
		RP11-468N22^a^	21q22.11-22.12
		WI2-1915K14^e^	*RUNX1* intron 1
		CTD-2349F18^a^	*RUNX1* intron1-exon2
		CTD-2235K24^a^	*RUNX1* exons 2–8
		WI2-942D2^e^	*RUNX1* exons 2–4
		WI2-605D9^e^	*RUNX1* exons 2–5
		WI2-847D7^e^	*RUNX1* exon 6
		WI2-542L2^e^	*RUNX1* exons 7-8
3	1	RP11-467K11^a^	1p36.32
		RP11-372C15^a^	1p36.31
		RP11-690E2^a^	1p36.23
		RP11-113C10^a^, RP11-90B12^a^	1p34.2, flanking the MPL gene
		RP11-46G23^a^, RP11-125P23^a^	1p12
		RP11-206H22^a^	1p11.2

^a^ BAC probes, Invitrogen Corporation, Carlsbad, CA, USA.

^b^ WCP, Cytocell Technologies, Cambridge, UK.

^c^ 21 qtel07, Cytocell Technologies, Cambridge, UK.

^d^ LSI AML1/ETO dual colour dual fusion translocation probe, Abbott, Abbott Park, IL, USA.

^e^ Fosmid probes, kindly provided by Prof. Peter De-Jong, BACPAC Resources Center, Childrens Hospital Oakland Research Institute, Oakland, CA, USA.

Patients (Pt), chromosome localization and sequences related to the genes of interest recognized.

**Figure 2:**
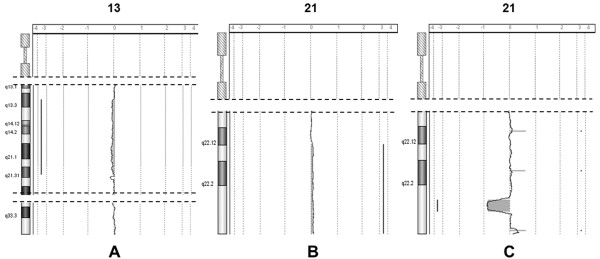
**a-CGH profiles of the regions of imbalance of patients 1 and 2.A**-**B**: Profiles of chromosomes 13 and 21 of patient 1 on DNA from BM sampled in June 2007; **C**: Profile of chromosome 21 of patient 2 on DNA from PB. The profiles shown were obtained with the 244 K genome-wide system

Array-based comparative genomic hybridization (a-CGH) was performed on DNA from BM sampled in June 2007, when the chromosome analysis showed one cell with the der(21), and seven with the int del(13) out of 20 mitoses; FISH on nuclei with the probes RP11-1001I7 (13q14.2), and CTD-2235K24 (*RUNX1,* exons 2–8) showed 11.5% (52/452) of BM cells belonging to the clone with the int del(13), and 9.3% (42/453) to the clone with the der(21). The size of these abnormal clones was above the limits of sensitivity for their detection that we had previously established [4]. The a-CGH profiles identified precisely the interstitial deletion as int del(13)(13q13.3-q21.31) with loss of more than 26 Mb (36 836 026 – 63 185 558 bp), and the 21q22.12-q22.3 duplication (10.3 Mb, 36 565 301 – 46 897 430 bp), but failed to detect the subtle deletion including the exons 2–8 of *RUNX1* (Figures 2A-B)*.*

**Figure 3:**
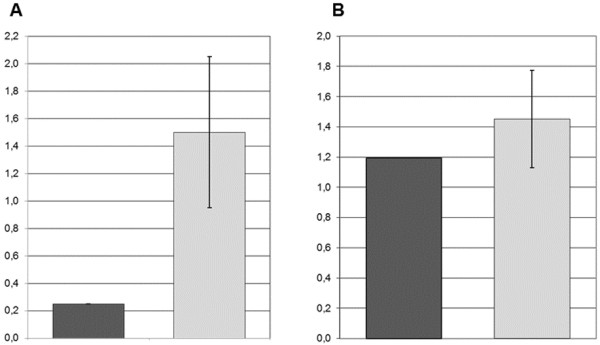
**Relative expression of *RUNX1 *in the BM of patient 1.** Results two years (**A**), and four years (**B**) after disease onset (2006 and 2008, respectively). The dark grey bars refer to the patient and the light grey bars to controls’ average values. Housekeeping control genes were *ACTB* in **A**, and *UBC* in **B**

The relative expression of *RUNX1* was evaluated on BM twice, after two and four years from disease onset, when the FISH on nuclei revealed the der(21) in 41% and 5% BM cells, respectively; the results showed a strong hypoexpression compared to the controls in the first assay (Figure 3A), and a less pronounced hypoexpression in the second one (Figure 3B).

### Patient 2

Cytogenetic investigations performed on BM, PHA-PB cultures, and skin fibroblast cultures revealed a constitutional rearrangement of chromosome 21 which had a submetacentric morphology suggesting a pericentic inversion, inv(21). The investigation by FISH with the library for WCP painted completely the inv(21), and the NOR-staining technique demonstrated one breakpoint to be inside the region 21p11.1-11.2, not involving the satellites. The FISH with the probe “21 qtel07” showed that the inversion did not involve the subtelomeric region, while FISH with “LSI AML1/ETO” showed two separate signals for *RUNX1*, one above and one below the centromere, indicating that the other breakpoint was localized inside the sequence recognized. We demonstrated that this breakpoint was between exons 4 and 5 inside *RUNX1*, being the signals related to the probes WI2-942D2 and WI2-605D9 present both in the short and long arms with variable and reduced intensity (Figure 1B). The probes WI2-1915K14, and CTD-2349F18 were present and not displaced by the inversion. The pattern of the pericentric inversion was further defined with the probes CTD – 2532E17, RP11 – 468N22, and with probes recognizing *RUNX1* exons 6 – 8 (Figure 1B).

**Figure 4:**
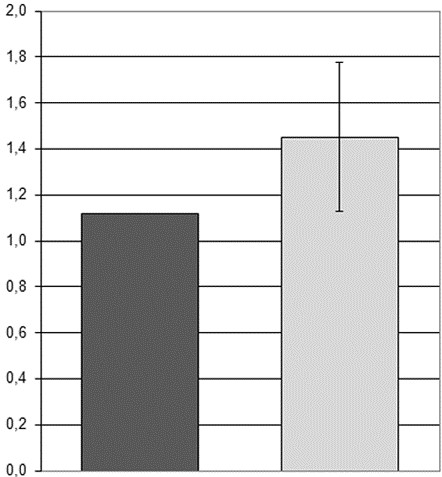
**Relative expression of *RUNX1 *in the BM of patient 2.** The dark grey bar refer to the patient and the light grey bar to controls’ average values, *UBC* was used as control

The a-CGH profile (Figure 2C) demonstrated four regions of imbalance, here described in order from the centromere to the telomere: 1) a duplication of 36.1 Kb inside the sequence of the disrupted *RUNX1* gene spanning from 35 138 169 bp to 35 174 269 bp, and including exon 5; 2) a duplication of 38 Kb (39 669 148 – 39 707 107 bp); 3) a deletion of 1.4 Mb (43 014 727 – 44 408 507 bp), confirmed by FISH with the lack of the signal for probe RP11 – 88 N2, (Figure 1B); 4) a duplication of 162 Kb (46 493 951 – 46 656 014 bp), shown to be a benign copy number variation (CNV) by the result of a-CGH performed with the parents’ DNA one vs. the other, inherited from the father and included in CNV Database of Genomic Variants, updated March 2010 [5]. Thus, the inv(21) resulted in fact in the disruption of the *RUNX1* gene, with a complex rearrangement including a tiny duplication of part of *RUNX1* itself, and two other imbalances, a deletion and a duplication, both more distal to *RUNX1*.

The relative expression of RUNX1 evaluated on BM sample was lower than controls (Figure 4).

### Patient 3

Repeated chromosome analyses performed on BM samples from August 2007 to March 2011 showed a clonal paracentric inversion of the long arms of chromosome 1, inv(1)(p13p36), as sole acquired anomaly. The anomaly was present in 12/15 cells at the first analysis (August 2007), then in the majority of the cells in repeated analyses from 2008 to 2011. PHA-PB cultures showed a normal karyotype (400 mitoses scored), and we ruled out definitely a constitutional mosaicism, as a normal karyotype was found also in fibroblasts from a skin biopsy, where it was confirmed by 200 nuclei analyzed by FISH with the probe RP11-372C15, which gave no signal in the inv(1), as better detailed below.

FISH was performed to investigate the breakpoints of the inversion and to look for the possible involvement of the *MPL* gene, localized in 1p34.2.

**Figure 5:**
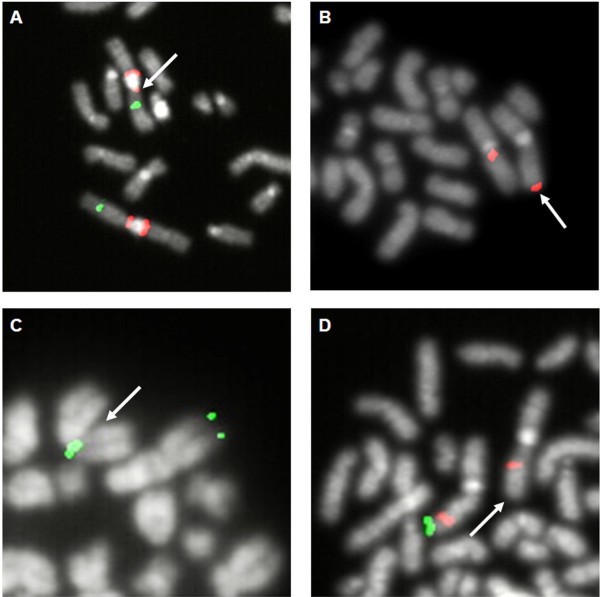
**Mitosis cut-outs with FISH results of patient 3.A**: the arrow indicates the signal of probe RP11-125P23 (red), at the normal localization, but smaller than the one on the normal 1; the red signal on the long arms, both on the normal 1 and on the inv(1), is due do cross-hybridization, and the green signal is the one of probe RP11-90B12, flanking the *MPL* gene, used as internal control and displaced towards the centromere on the inv(1); **B**: the arrow indicates the signal of probe RP11-46G23 (red), moved towards the telomere on the inv(1); **C**: the arrow indicates the signal of probe RP11-690E2 (green), moved towards the centromere on the inv(1); **D**: the arrow indicates the inv(1) lacking the signal of probe RP11-372C15 (green, on the normal 1); the red signal is the one of probe RP11-113C10, flanking the *MPL* gene, used as internal control and displaced towards the centromere on the inv(1)

**Figure 6:**
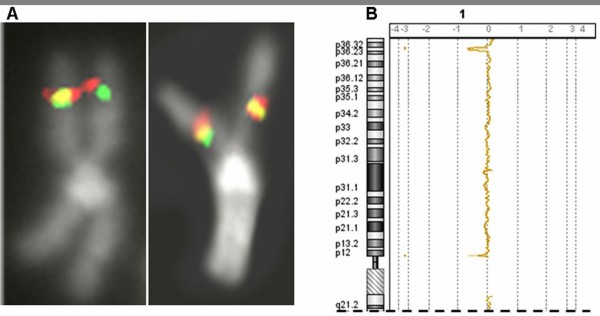
**Patient 3: FISH for *MPL *gene and a-CGH results.** Probes flanking the *MPL* gene indicate its localization on the inverted chromosome 1, at the right (**A**); a-CGH profile of the short arms of chromosome 1 shows the two deleted regions (**B**)

The proximal breakpoint was inside the band 1p12 because the results were the following: the signal of the probe RP11-206H22 (1p11.2) was at the normal position, as was the one of the probe RP11-125P23 (1p12), which appeared smaller compared to the normal 1 (Figure 5A), whereas the signal of the probe RP11-46G23 (1p12) was moved towards the telomere (Figure 5B). The distal breakpoint was between bands 1p36.23 and 1p36.32, as the signal of the probe RP11-467K11 (1p36.32) remained in the expected localization, whereas the one of the probe RP11-690E2 (1p36.23) was displaced towards the centromere (Figure 5C). The probe RP11-372C15 (1p36.31), in the region of the distal breakpoint failed to show any signal in the inv(1) (Figure 5D). Among the results listed, two findings were unexpected: the smaller signal of the probe RP11-125P23 (1p12), and the lack of the sequence recognized by the probe RP11-372C15 (1p36.31). The possible involvement of the *MPL* gene was studied by FISH with two flanking probes, (RP11-113C10 and RP11-90B12), that demonstrated that the gene was not disrupted, in a position closer to the centromere than normal (Figure 6A).

**Figure 7:**
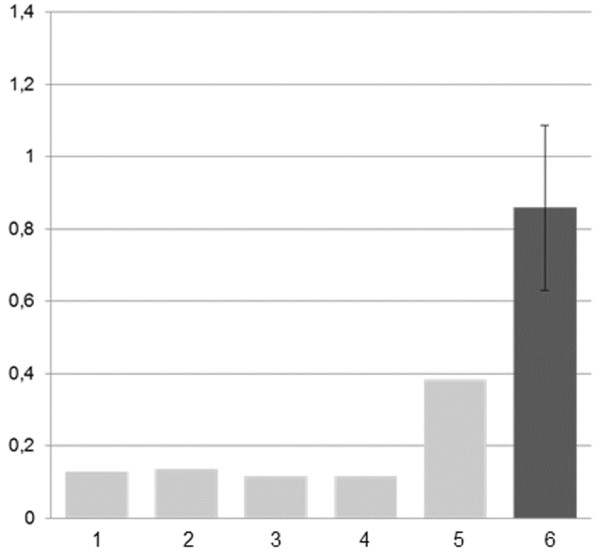
**Patient 3: relative expression of *MPL *in BM.** BM sampled at five different dates (light grey columns 1–5), compared to five control subjects (dark grey column 6: mean value ± standard deviation)

The a-CGH results explained the unexpected FISH findings, showing two deleted regions: a segment of 1.65 Mb in 1p36.31 (5 390 817 – 7 024 313 bp), and one of 350 Kb in 1p12 (120 152 971 – 120 495 484 bp) (Figure 6B). The relative expression of *MPL* was repeatedly evaluated on BM sampled in April 2008, March 2009, October 2009, February 2010, and June 2010, and it was always strongly reduced in comparison to controls (Figure 7).

## Discussion

The *RUNX1* gene is involved in several different and frequently recurring translocations with various partner genes in leukaemia, and point mutations or deletions of this gene are relevant as well in the pathogenesis of different types of MDS and leukaemia subtypes [6-9]. Germline heterozygous mutations of *RUNX1* cause the autosomal dominant disease “familial platelet disorder with propensity to AML” (FPD/AML) (OMIM #601399). Therefore, it is not surprising that *RUNX1* haploinsufficiency due to constitutional chromosome deletions may cause a clinical phenotype which includes thrombocytopenia besides intellectual disability and other symptoms [10]. Only one case is reported in the literature with a constitutional *RUNX1* deletion leading to non-syndromic thrombocytopenia with MDS [11]. Patients 1 and 2 reported here demonstrate that thrombocytopenia or BM hypoplasia with consequent SAA may in fact be due to structural anomalies of chromosome 21 involving *RUNX1* which are not deletions, but complex rearrangements which may also be acquired instead of constitutional.

In detail, the chromosome anomaly in patient 1 was acquired and clonal in BM as shown by chromosome analyses and FISH on interphase nuclei. The fact that the der(21) was already present at the onset of the SAA provides support for its relevance in the disease aetiology. Altogether, our results demonstrate that the der(21) implies a deletion of most of the *RUNX1* gene, besides the inverted duplication of a more distal 10.332 Mb region (Figures 1A-2B): the expression of RUNX1 is consequently reduced (Figure 3) and causes defective maturation/proliferation of haematopoietic cells and SAA, what does not exclude the possible action also of other concomitant factors. During the disease course the abnormal clone varied in size within a range from 5% to 50%, as shown from chromosome and FISH analyses, without any specific clinico-haematological variation. *RUNX1* expression in BM varied in parallel with the different size of the clone with the der(21) (Figure 3). Since November 2006 to November 2008, this abnormal clone decreased in size (to 9%-5%) at the same time of the appearance of a new independent clone with the int del(13)(q12-13q21) which, on the contrary, increased progressively in size (5%-32%). Worthy of note, similar deletions of chromosome 13 are recurrent in myeloproliferative disorders, in particular in polycythaemia vera and primary myelofibrosis, but also in MDS and AML [12,13]: no morphological signs of overt MDS, however, was ever noticed in our patient 1.

In patient 2 the complex rearrangement of chromosome 21 led to three regions of imbalance, two duplications and one deletion, besides the benign CNV inherited from the father. The *RUNX1* gene was disrupted by the rearrangement, and a tiny segment including exon 5 was duplicated. During the diagnostic procedures, a mutation analysis of *RUNX1* had been performed, but failed to reveal any change, obviously because no mutation was present in exon sequences. So, in this patient the disruption of *RUNX1* led to decreased expression (Figure 4) and to thrombocytopenia. Being the rearrangement constitutional, we searched accurately for possible clinical findings, other than haematological, which might be related to the imbalances: when she was 8-year-old, height and weight were between the 25^th^ and the 50^th^ centile, there was no developmental delay neither mental retardation, and none of the dysmorphic/malformative signs described in cases of 21q22 overlapping deletions [14], or any other relevant symptoms, were present.

The definite diagnosis of patient 3 is CAMT. CAMT is an autosomal recessive disorder characterized by absent or reduced number of Mks in the BM since birth, extremely elevated serum levels of TPO, and very low platelet count that in some cases increases transiently during the first year of life [15]. Prognosis of CAMT patients is poor, because all of them develop in childhood a tri-linear marrow aplasia that is always fatal when left untreated. In our patient, pancytopenia, severely reduced BM cellularity, increased TPO levels and reduced platelet expression of TPO receptor, suggested a diagnosis of CAMT. However, homozygous or compound heterozygous mutations of the *MPL* gene, coding for the TPO receptor, that are responsible for CAMT [15], were not identified: nevertheless, expression analysis showed a very low level of *MPL* transcript (Figure 7). Moreover, polymorphism analysis showed that the patient had inherited from the parents the same alleles of his healthy sister.

The locus *MPL* is on the short arms of chromosome 1, in band 34.2, and the BM clonal structural anomaly just of the short arms of chromosome 1 may hardly be considered as fortuitous. FISH with probes flanking *MPL* and a-CGH results excluded the disruption of the *MPL* gene (Figure 6).

We postulate the following pathogenetic mechanism for CAMT in our patient. The paracentric inversions are usually considered balanced anomalies, but it has been shown that a-CGH applied to search for constitutional anomalies is able to detect a number of imbalances undetected with conventional cytogenetic methods, as was the case, e. g., of 10% of 13,926 patients with mental disability and apparently normal karyotype reviewed by Sagoo et al., 2009 [16]. In patients with apparently balanced constitutional structural rearrangements, it has been demonstrated that many cryptic deletions may be found by a-CGH, more frequently at the breakpoint, with consequent phenotypic abnormalities [17]. So, the finding of two cryptic deletions at the breakpoints of the inversion of our patient is not too surprising: it is conceivable that this kind of unexpected imbalances may be found in acquired anomalies as well, and that position effects may derive from these cryptic imbalances. A well-known example of these cryptic rearrangements in an acquired apparently balanced translocation, regards chronic myeloid leukaemia with the Ph chromosome by translocation t(9;22)(q34;q11), in which deletions in regions flanking the translocation breakpoints are frequent, and imply a poor outcome [18]. In constitutional anomalies, firm evidence is available of the deregulation of transcripts due to the dissociation from long-range regulatory elements, phenomenon usually called position effect: structural anomalies were shown to be able to act on specific genes which may be tens of megabases apart from the breakpoints identified [19,20]. The a-CGH results in patient 3 showed a distance of about 36.5 Mb from the *MPL* gene to the telomeric breakpoint of the inversion and of about 76.5 Mb to the centromeric one. The evidences from the literature on constitutional rearrangements concern position effects acting even at distance, but always on the chromosome where the breakage has took place, in *cis*, not on its homologue, but in mammals long-range DNA interactions were demonstrated acting both in *cis* and in *trans*, and causing variable gene expression level [21]. So, we postulate that in patient 3 the primary event causing the disease was the acquired clonal paracentric inversion, with the loss of the two regions near the breakpoints, and that some sequences in the lacking segments impaired the function of the *MPL* gene by position effect, both on the inv(1) and on the normal 1, thus leading to highly reduced expression and to acquired CAMT.

## Conclusions

The three cases here reported lead to the following conclusions as to the role of chromosome changes in the pathogenesis of peripheral cytopenias and BM hypoplastic conditions: 1) the presence of an acquired clonal anomaly in BM does not suggest *per se* MDS in patients with inconclusive morphologic features: a subgroup of BM hypoplastic disorders, with uni- or multi-lineage effects, is directly due to a chromosome structural anomaly in BM which causes a specific genic effect. This pathway might be rather frequent: we have on record at least another case of thrombocytopenia which is due to a similar mechanism, an adult patient with an acquired complex chromosome rearrangement in BM implying deletion of long arms of chromosome 11 with loss of the *FLI1* gene, and causing a thrombocytopenia of the Paris-Trousseau type [22]. 2) Structural anomalies of chromosome 21 may impair *RUNX1* expression by deletion or simple disruption of the gene, as in patients 1 and 2, and they may be either acquired or constitutional. 3) The chromosome anomaly may act without deletion or disruption of the gene, but by impairing the gene expression, as is the case of *MPL* in our patient 3.

The clinical “take-home message” of our report is that comprehensive cytogenetic investigations, if possible including a-CGH, should always be performed for the diagnostic evaluation of patients with BM aplasia/hypoplasia and peripheral cytopenias: in this regard, it is worth noting that the reduced *RUNX1* expression in the abnormal BM cells of patients 1 and 2 is strictly comparable to that of patients with FPD/AML, in whom a 20-50% risk of MDS/AML is expected [23].

## Methods

Chromosome analyses were performed in the three patients with routine methods and QFQ-banding technique on BM direct preparations and 24-48^h^ cultures, on PB unstimulated and PHA-stimulated cultures, and, in patients 2 and 3, also on fibroblasts cultured from a skin biopsy. In patient 2, also NOR-staining technique was applied.

FISH on metaphases, and on interphase nuclei was done by standard procedures with different probes to define the chromosome anomalies, and to monitor the abnormal clones. All the probes used for each patient in the FISH assays are listed in Table 1.

The a-CGH was performed with the 244 K genome-wide system (Agilent Technologies Inc., Santa Clara, CA, USA), according to the manufacturer’s instruction on DNA from BM sampled in 2007 of patient 1, on DNA from PB of patient 2 and her parents, and on DNA from BM sampled in November 2008 of patient 3.

The DNA was extracted using the Qiagen Blood and Tissue kit (QIAGEN GmbH, Hilden, Germany), and competitor DNA was purchased from Promega (Promega Corporation, Madison, WI, USA). Slides were scanned using Agilent’s microarray scanner G2565CA and microarray images were analysed using Agilent’s Feature Extraction 10.7.3.1 software, and by Agilent’s Genomic Workbench software (5.0.14). All map positions in the results refer to the genome assembly hg18.

In patients 1 and 2 the relative expression of the *RUNX1* gene was evaluated on RNA from total BM using Applied Biosystems ABI 7000 real-time thermocycler (Life Technologies Corporation, Carlsbad, California, USA), and the results were compared with RNA from BM of 4 age-matched healthy control subjects who donated haematopoietic cells for transplantation of a relative. In patient 1 this assay was performed twice, after two and four years from onset (2006, 2008), with Applied Biosystems Taqman assay # Hs_00231079_m1 for *RUNX1*, and with endogenous controls which were # Hs_99999903_m1, Actin beta gene (*ACTB*), in the first assay, and # Hs_00824723_m1, Ubiquitin C (*UBC*) in the second one. Patient 2 was examined with the same technique with Applied Biosystems Taqman assay # Hs_00231079_m1 for *RUNX1*, and # Hs_00824723_m1, *UBC*, as endogenous control.

In patient 3, the relative expression in BM of the *MPL* gene was repeatedly evaluated on RNA from total BM, sampled at five different dates from April 2008 to June 2010, using the same thermocycler as above, with Applied Biosystems Taqman assay # Hs_00180489_m1 for *MPL*, and the housekeeping gene *HPRT1*, Taqman assay # Hs_01003267_m1, as normalizer: the results were compared with RNA from BM of 6 age-matched healthy control subjects.

Informed consent to this study was obtained according to the principles of the Declaration of Helsinki from patients’ parents, and healthy controls; the experimental work was approved by the Ethical Committee of Fondazione IRCCS Policlinico S. Matteo, Pavia.

## Competing interests

The authors declare that they have no competing interests.

## Authors’ contributions

CM, BP, LM and GMo contributed equally to chromosome analyses and FISH. CM and RV performed array-CGH analyses and mutational analysis of patients 1 and 2. GMe, GL, MEB, LV, ADC-M, MZ and FLo were responsible for the clinical management of the patients and of the analysis of clinico-haematological data. SF performed molecular analysis of patient 3. FL, FLo, FP and EM conceived and coordinated the study, and drafted the manuscript. All authors have read and approved the final manuscript.
